# Effect of Glycated Haemoglobin (HBA1c) on Cardiorespiratory Fitness (CRF) in a Population with Type 2 Diabetes Mellitus (T2DM): A Cross-Sectional Study

**DOI:** 10.3390/medicina60111823

**Published:** 2024-11-06

**Authors:** Snehil Dixit, Daniela Bassi-Dibai, Almir Vieira Dibai-Filho, Renata G. Mendes, Abdulfattah S. Alqahtani, Mohammed M. Alshehri, Monira I. Aldhahi, Batool Abdulelah Alkhamis, Ravi Shankar Reddy, Jaya Shanker Tedla, Audrey Borghi-Silva

**Affiliations:** 1 Department of Medical Rehabilitation Sciences, College of Applied Medical Sciences, King Khalid University, Abha, Alfara 61421, Saudi Arabia; 2 Postgraduate Program in Management and Health Care, Ceuma University, São Luís 65075-120, MA, Brazil; 3 Postgraduate Program in Physical Education, Federal University of Maranhao, São Luís 65080-805, MA, Brazil; 4 Cardiopulmonary Physiotherapy Laboratory, Physiotherapy Department, Federal University of Sao Carlos, Sao Carlos 13565-905, SP, Brazil; 5 Department of Rehabilitation Health Sciences, College of Applied Medical Sciences, King Saud University, Riyadh 11451, Saudi Arabia; 6 Department of Physical Therapy, Faculty of Applied Medical Sciences, Jazan University, Jazan 45142, Saudi Arabia; 7 Department of Rehabilitation Sciences, College of Health and Rehabilitation Sciences, Princess Nourah bint Abdulrahman University, Riyadh 11671, Saudi Arabia

**Keywords:** cardiorespiratory fitness, type 2 diabetes mellitus, glycaemic control, diabetic complications, exercise capacity, glycated haemoglobin

## Abstract

*Background and Objective:* The aim of this study was to evaluate cardiorespiratory fitness (CRF) measures, maximal oxygen consumption (VO_2_ max), and minute ventilation/carbon dioxide production (V_E_/VCO_2_ slope and others) among the T2DM population based on glycated haemoglobin (HBA1c). *Material and Methods:* The present study comprised a cross-sectional design, with two groups, based on HbA1c values (≤7 and ≥7.1). Laboratory samples were taken to evaluate glycated haemoglobin and fasting blood glucose (FBS). Cardiopulmonary exercise testing was performed to calculate various fitness-related parameters. Data analysis: An independent *t*-test was used to analyse the outcomes in the two groups. *p* < 0.05 was considered significant. Linear regression was used to examine the influence of predictor variables on dependent variables. *Results:* A total of 70 patients agreed to participate in the study, with 19 females and 51 males. The mean (standard deviation) BMI (body mass index) of all participants was 29.7(5.2), the mean (SD) weight was 84.4 (18.9) kg, and the mean height was 167.4 (23) cm. The average age of the individuals was 52 ± 8 years. The independent *t*-test revealed a significant difference between the two groups in terms of CRF measures. *Conclusions:* The current research identified the presence of poor glycaemic control and cardiorespiratory fitness measures among the Brazilian population with T2DM. HBA1c, duration of diabetes, age, and BMI can be employed to predict the ventilatory threshold (VT) and VO_2_ max.

## 1. Introduction

Type 2 diabetes mellitus (T2DM) is a chronic metabolic disorder that manifests as a continuous state of hyperglycaemia. T2DM is primarily due to advanced loss of adequate insulin-secreting beta cells, due to a constant state of insulin resistance [1]. The worldwide prevalence of T2DM is forecasted to increase to 7079 individuals per 100,000 by 2030, with emerging trends of increasing prevalence among less developed countries. In Brazil, the rough incidence rate of T2DM among males increased by 48% during 1990–2017, and among females increased by 49%. The prevalence among males increased by 30%, with an increase in females of 26% during the same period [2]. 

According to the Centers for Disease Control and Prevention (CDC), some racial and ethnic communities are at a higher risk for T2DM. The high-risk populations include Asian Americans, Hispanic Americans, and African Americans [2]. The risk of diabetes is constantly increasing due to genetic configurations, eating habits, and physical inactivity [2]. The HBA1c levels in T2DM patients are often associated with micro- and macrovascular complications, with a high risk of mortality among young obese individuals [3]. Higher concentrations of HBA1c in the body may be associated with adverse cardiovascular events and the risk of mortality [3]. Moreover, there is evidence indicating that T2DM is associated with higher rates of mortality, morbidity, and reduced quality of life [4].

Diabetes affects individuals’ functional capacities and cardiorespiratory fitness (CRF), parameters that are important indicators of the health status [5]. Poor CRF is often associated with an increased risk of cardiovascular events and death in T2DM patients and healthy youths [5,6]. There is mounting evidence from epidemiological and clinical research indicating that CRF is a robust predictor of mortality compared to other established risk elements such as hypertension, hypercholesterolemia, smoking, and T2DM [7]. 

Recent research has outlined the strength of the association of T2DM with the risk of mortality. The risk of mortality is seemingly higher among high-income economies than mid- and low-income economies [4]. An inactive lifestyle featuring physical inactivity among the T2DM population further complicates the issue. The American Diabetes Association (ADA) recommends that people with diabetes should perform aerobic exercise. In an ideal world, an aerobic exercise session should last for 10–30 min/day and be undertaken on most days in the week for an adult with T2DM [8]. Cardiorespiratory fitness through exercise provides a feeling of well-being, improve weight control, and aid in glucose control. Regular physical activity can have a substantial effect on the overweight population in maintaining weight loss [9].

Consistent physical activity is linked to improved cardiorespiratory fitness and a decreased risk of chronic illnesses, such as cardiovascular disease and death [10]. In a long-term follow-up study, a comprehensive analysis was conducted on several factors, including age, systolic blood pressure, smoking status, VO_2_ max, low- and high-density lipoprotein cholesterol and triglycerides, body mass index, C-reactive protein, physical activity, alcohol consumption, socioeconomic status, history of ischemic heart disease, and type 2 diabetes. The study concluded that long-term CRF decline was associated with a greater risk of mortality [11].

Diabetes has been linked with untimely deaths from cardiovascular disease, cancer, and non-cardiovascular and non-cancer origins. Therefore, the assessment of CRF among T2DM individuals is of utmost importance [12]. The life expectancy of a person 50 years of age with T2DM is supposedly six years less than it would be without T2DM [13]. Diabetes quadruples the cardiovascular risk among affected individuals compared to the unaffected population [14]. In addition, T2DM increases the risk of cancer, as shown by several cohort studies [15]. In this context, controlling glycaemic indexes over time can identify patients at risk of all fatal and non-fatal outcomes.

However, there is a dearth of studies in the literature regarding the effect of higher HBA1c levels on cardiorespiratory fitness (CRF) parameters in T2DM individuals in the Brazilian population. Hence, the objectives of the present study were to evaluate the impact of glycaemic control (HBA1c) based on HBA1c levels < 7 (group 1) and >7.1 (group 2) on cardiorespiratory fitness parameters among the T2DM population in Brazil. 

## 2. Materials and Methods

### 2.1. Design

**Figure 1:**
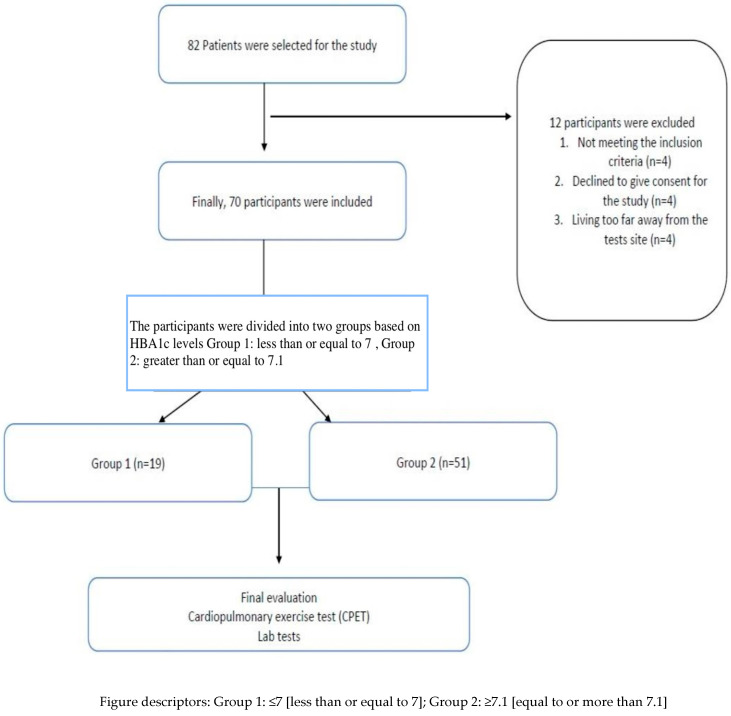
Flow chart for participants in this study.

This was a cross-sectional study in which the population was divided into two groups based on HBA1c values (≤7 (group 1; G1) and ≥7.1 (group 2; G2)) to examine the effects of glycaemic control on CRF parameters. There were 19 participants in group 1 and 51 participants in group 2. The flow chart for the patients is shown in Figure 1. 

This research was conducted as per the Declaration of Helsinki guidelines and was approved by the Human Research Ethics Committee of the Institute, Federal University of Sao Carlos (protocol number 1318/1). 

The participants who were diagnosed with T2DM were recruited from the cardiovascular outpatient clinic of the Federal University of Sao Carlos (UFSCar). The durations of T2DM and other demographic variables were recorded. The research was performed in the UFSCar Cardiopulmonary Physiotherapy Laboratory. 

Inclusion criteria for both groups consisted of diagnosed cases of T2DM based on fasting blood glucose (FBS) and HbA1c values. An inactive lifestyle for at least six months was assessed according to the guidelines recommended by the American Heart Association (AHA) for sedentary behaviours [16]. All patients at the start of this study were assessed as sedentary (self-reported). Exclusion criteria were a history of recent revascularisation surgery within one month, previous acute heart disease, high-risk patients, neurological or cognitive dysfunction affecting the ability to understand instructions, and musculoskeletal dysfunction limiting the ability to perform exercise testing. 

### 2.2. Laboratory Samples 

HbA1c was evaluated by anion-exchange high-performance liquid chromatography (Variant II, Bio Rad, Berkeley, CA, USA) at the central laboratory coupled with a fluorescence indicator recommended by the National Glycohemoglobin Standardization Program [17]. Evaluation of fasting plasma glucose was performed by an enzymatic method using an AU 680^®^ (Beckman Couter, Suarlée (NAMUR), Belgium), and a chemiluminescent assay (UniCel^®^ DxI 800, Pasadena, CA, USA) was used to evaluate fasting plasma insulin [18]. Samples were acquired after an overnight fasting period.

### 2.3. Cardiopulmonary Exercise Testing (CPX)

Before the actual test day, the participants were taken to the research room for familiarisation. The participants were instructed to avoid the following: (1) caffeinated or alcoholic beverages consumed the previous night and on the actual allotted day of the test; (2) moderate-intensity to high-intensity physical activity on the day of examination [18]. 

Incremental exercise testing was conducted using a cycle ergometer (Recumbent Corival of MedGraphics, Namur, USA) using a gas exchange method that was symptom limited. The ventilatory variables were calculated by a well-calibrated computerised exercise assessment system (Metabolic analyser System Greenhouse telemetry module for field studies Oxycon-Mobile, Jaeger, Hoechberg, Germany). 

The exercise test was divided into several stages, with five minutes of rest followed by four minutes of exercise with no workload and then an incremental phase with one minute of active recovery and five minutes of passive recovery [18]. The load (W) was increased linearly; for the “ramp” protocol of 15 W min^−1^, the duration for incremental exercise evaluation was 8 to 12 min. The exercise test was terminated based on the following criteria: (1) achievement of a respiratory exchange ratio (RER) ≥ 1.10; (2) attainment of age-predicted maximal heart rate (HR) ≥ 85%; (3) physical symptoms such as dyspnoea or fatigue and/or inability to maintain a sufficient work rate due to equipment limitations. When visible signs of physical fatigue were observed among the participants or if there was an abnormal test response, the evaluation was terminated [18]. 

During the test, the mean value of the maximum during fifteen seconds was recorded as the VO_2_ max. The values obtained for fifteen seconds were summed for ventilation (V_E_) and carbon dioxide production (VCO_2_) and were plotted in an Excel spreadsheet (Microsoft Excel, version 1997–2004, Microsoft Corp., Bellevue, WA, USA). Then, the values for V_E_ and VCO_2_ during the evaluation were utilised to estimate the V_E_/VCO_2_ slope [18]. The oxygen uptake efficiency slope (OUES) was calculated using the linear relationship between VO_2_ (mL/min) and V_E_ (L/min), through the equation Y = ax + b, where y = VO_2_; a = VO_2_ increase rate in response to V_E_ increase; b = intercept, defined by VO_2_ at rest; x = VE, transformed into LOG in 10 base due to the non-linear behaviour of VE (L/min) during maximum effort. The OUES was analysed from the beginning of the exercise to the maximum effort (OUES100%) [19]

### 2.4. Data Analysis 

The analysis was performed using SPSS version 24. The mean and standard deviation (SD) were calculated for all continuous variables. An independent *t*-test was used to compare the two groups (based on HBA1c levels < 7 and >7.1) and analyse the changes in the outcomes in terms of CRF variables. *p* < 0.05 was considered significant. The analysis was carried out using the following parameters: peak VO_2_ (mL/kg/min), V_E_/VCO_2_ slope, VT, fasting blood sugar (FBS), and HBA1c. Levene’s test for equality of variances was used to interpret whether equal variance was assumed. Additionally, the normality of distribution was interpreted using a histogram; if the data were skewed, then equality in variance was not assumed. If the variance was violated, then the result was interpreted with equal variance not assumed. Results were reported for each parameter using the F value, significance value (*p* < 0.05), T value [degrees of freedom (Df)], and F value with a 95% confidence interval of difference. Linear regression equations were constructed to analyse the impact of independent variables on dependent variables. 

## 3. Results

A total of 70 patients agreed to participate in the current study. The study population comprised 19 females and 51 males. A flow chart for the participants is presented in Figure 1. The overall mean (SD) BMI (body mass index) of the participants was 29.7(5.2), the mean weight in kilograms (kg) was 84.4 (18.9), and the mean height (centimetres) was 167.4 (23) cm. The demographic details of the T2DM participants from the Brazilian population are listed in Table 1. The overall mean age and standard deviation of the participants in the study was 52 ± 8 years. Four participants were on drugs such as angiotensin-receptor blockers/angiotensin-converting enzyme inhibitors, five were on beta-blockers, and 10 were on diuretics.

An independent *t*-test was used to compare the two groups (based on HBA1c < 7 and >7.1) using the following parameter: peak VO_2_, V_E_/VCO_2_ slope, VT, OUES, peak RER, and fasting blood sugar (FBS). 

The independent *t*-test for peak VO_2_, on inspection of the data, was normally distributed for both the groups, and there was homogeneity of variance, as evaluated by Levene’s test for equality of variances. Therefore, it was found that peak VO_2_ in the group where HBA1c levels were less than 7 [Mean (SD)] = 22.35(5.23), and for HBA1c > 7 = 21.05(5.04) [F = 0.33, t (66) = 0.91, *p* = 0.57], with a mean difference of 1.3 with the CI at 95% [−1.6–4.15]. 

Upon examining the histogram for the fasting glucose, the data for both groups were normally distributed, and Levene’s test for equality of variances indicated that there was homogeneity of variance. It was found that fasting glucose in the group where HBA1c levels were less than 7 [Mean (SD)] = 123(31.6), and for HBA1c > 7 = 164(60.03) [F = 4.41, t (53) = −2.74, *p* = 0.04*], with a mean difference of −41.9 with the CI at 95% [−72.4–−11.4]. 

The data for both groups were normally distributed for the ventilatory threshold upon analysis, and Levene’s test for equality of variances also indicated homogeneity in variance. It was found that the ventilatory threshold in the group where HBA1c levels were less than 7 [Mean (SD)] = 14.3(5.15), and for HBA1c >7 = 13.4(3) [F = 4.5, t (32) =0.56, *p* = 0.04*], with a mean difference of 0.81 with the CI at 95% [−2.1–3.74]. 

For the VE/VCO_2_ slope, on inspection of the data, it was not normally distributed for both the groups, as evaluated by Levene’s test for equality of variances; hence, equal variance was not assumed. It was found that the VE/VCO_2_ slope in the group where HBA1c levels were less than 7 [Mean (SD)] = 32.2(6.7), and for HBA1c > 7 = 33(8) [F = 0.07, t (32) = −0.43, *p* = 0.04*], with a mean difference of −0.84 with the CI at 95% [−4.8–3.2].

The OUES revealed that the data for both groups were normally distributed, and the homogeneity of variance was demonstrated by Levene’s test for equality of variances. It was found that the OUES in the group where HBA1c levels were less than 7 [Mean (SD)] = 2.14(0.6), and for HBA1c > 7 = 2(0.6) [F = 0.31, t (66) = −0.79, *p* = 0.6], with a mean difference of 0.14 with the CI at 95% [−0.21–0.48]. 

Our analysis of peak RER revealed that, according to Levene’s test for equality of variances, the data for both groups were not normally distributed; thus, equal variance was not assumed. It was found that peak RER in the group where HBA1c levels were less than 7 [Mean (SD)] = 1.05(0.24), and for HBA1c > 7 = 2(0.1) [F = 5.4, t (17) =−0.69, *p* = 0.02*], with a mean difference of 0.04 with CI at 95% [−0.85–0.17].

Analysis of the heart rate recovery in one minute showed that equal variance was not met, with the data for both groups not normally distributed, as indicated by Levene’s test for equality of variances. It was found that heart rate recovery in the group where HBA1c levels were less than 7 [Mean (SD)] = 76.53(66.5), and for HBA1c > 7 = 73.6(60.6) [F = 1.33, t (26) =−0.16, *p* = 0.25], with a mean difference of 0.04 with CI at 95% [−34.6–40.54]. 

A Pearson correlation analysis was performed to examine the degree of association between glycaemic variables and CRF variables (Table 2). The stratification model using VO_2_ max for interpretation and risk stratification is presented in Table 3. The model used for the interpretation of outcome variables was taken from a previous study by Almeida et al. [20], and risk stratification was derived from the recommendations as stipulated by the Brazilian cardiovascular rehabilitation organisation [21].

The model used for interpretation was derived from Almeida et al. [20], and risk stratification was derived from the Brazilian cardiovascular rehabilitation guidelines [21].

### Linear Regression Models 

Linear regression between the independent variables the duration of diabetes, age, and HBA1c range (<7 and >7.1) and the dependent variable peak HR during the test showed a significant regression (Df = (3,62), F = 4.33, *p* = 0.008, R^2^ = 17.3). 

Linear regression models were employed to predict the dependent variable ventilatory threshold (VT) using the independent variables HBA1c, duration of diabetes, age, and BMI. A significant regression was found (Df = (4,61), F= 1.3, *p* = 0.004, R^2^ = 0.22). The measurement of VT changed by 0.22 (22%) with each unit change in the predictor variables. 

A linear regression model was also created to predict the dependent variable VO_2_ max as a function of the independent variables HBA1c, duration of diabetes, age, and BMI. A significant regression was found for BMI and VO_2_ max (Df = (3,64), F = 3.7, *p* = 0.02, R^2^ = 0.15). The measurement of VO_2_ max changed by 0.15 (15%) for a unit change in the predictor variable.

## 4. Discussion 

Diabetes is the ninth-leading cause of death worldwide [22]. According to a report released by the World Health Organization (WHO), the causes of death can be grouped into three categories: injuries, communicable diseases, and non-communicable diseases [22]. Previous research studies have uncovered an inverse relationship between CRF and T2DM, and the former has emerged as the single most important risk factor contributing to the increase in T2DM as well as the evolution of CVD in T2DM [23,24]. There are only a few studies that have examined the causal relationships that exist between HBA1c (<7 and >7) and CRF. 

The results of the current study are in agreement with the observation of poor CRF among the T2DM Brazilian population compared to an age-matched healthy Brazilian population [20]. We also observed that the variance between the two groups was significantly different in terms of V_E_/VCO_2_ slope. Previous research has established a prognostic value of V_E_/VCO_2_ slope for cardiac-related occurrence in those with a pre-existing history of heart failure [25]. The risk of mortality is greater when the V_E_/VCO_2_ slope exceeds 32.8 [25]. An abnormality in VO_2_ max or the V_E_/VCO_2_ slope may also reflect primary myocardial impairments among patients with T2DM [26]. According to a recent report, cardiovascular disease risk and mortality are increased in T2DM patients [27]. In contrast, improved CRF is linked with reductions in CVD, risk of cardiovascular death, and transience in men with T2DM [27]. 

Moreover, in T2DM that is uncomplicated, where individuals are otherwise asymptomatic, effort intolerance and a lowered VO_2_ peak are a major and frequent presentation. It is postulated that this occurs as a result of a significant flaw in the oxygen extraction process in the skeletal muscle and due to mild failure of the myocardial systolic pump [28]. Still, in the present study, the variance between the two groups was not significantly different in terms of peak VO_2_.

However, CRF in T2DM is a modifiable risk factor [29]. A previous census indicated that people with T2DM had a greater chance of developing health-related problems, including chronic kidney disease (CKD), cardiovascular disease (CVD), and congestive heart failure (CHF), with increased morbidity and mortality [29]. In addition, premature CVD-related deaths are 2- to 6-fold higher in T2DM patients than in those without T2DM [30]. The rate of CVD-related deaths in the T2DM population is twice that of other age-matched individuals. Chronic heart disease (CHD), stroke, sudden death, angina pectoris, and myocardial infarction (MI) are twice as common among the population suffering from T2DM as in the non-diabetic population [31]. 

In the present study, we demonstrated the existence of poor cardiorespiratory fitness among the Brazilian population with T2DM. The ventilatory threshold in the present study was significantly different between the two groups, signifying a better value with good glycaemic control. This study also highlights that with increasing BMI, the CRF parameters were further decreased, emphasising the need for weight management in controlling the complications of T2DM. A meta-analysis examined the effect of BMI on T2DM and found that mortality among the population with T2DM diminished with a BMI up to 28 kg/m^2^ but increased when the BMI exceeded 30 kg/m^2^ [32]. A research study also reported that HbA1c affects the VO_2_ peak and ventilatory threshold in obese and overweight subjects. The study found that even a slight deterioration in glycaemic management can impair cardiovascular health [33].

Peak exercise oxygen uptake (VO_2_ max) is a precise and consistent measure for estimating exercise capacity [34]. In a previous research study, subjects with T2DM were evaluated at baseline and then were followed up for five years for the occurrence of cardiovascular disease. The individuals who developed CVD issues during the five-year follow-up had a significantly lower baseline peak VO_2_ max [34]. Diabetes patients with poor glycaemic control experience earlier cellular involvement. The energy metabolism of the cells is impacted by HbA1c’s high affinity for oxygen, and there is a clinical link between persistent fatigue and declining exercise ability [35].

Population-based trials have revealed that hyperglycaemia can play a vital role in the development of CVD [36]. Chronic hyperglycaemia may lead to microvascular and macrovascular complications in a chronic situation. Patients with undiagnosed T2DM and chronic T2DM are at a higher risk of macrovascular syndromes such as peripheral vascular diseases, coronary heart disease (CHD), and stroke [36].

RER is another index that is used in the interpretation of results from exercise testing. The measure is considered to be less sensitive than other CRF measures [37]. In the present study, the two groups demonstrated significant difference in peak RER. The reason could be better glycaemic control in the group with HBA1c less than 7. The submaximal range of RER is estimated to be between 0.7 and 1.1, whereas during maximal exercise, the range is greater than 1.1 [37]. A higher value of RER indicates the dominance of carbohydrate metabolism, whereas low RER values are associated with a dominance of lipid oxidation [38]. 

Dietary intervention is important for achieving optimal blood glucose control. A glycaemic index of dietary items may help to achieve glucose control within the desired limit [39]. Structured employment of physical activity can play a vital role in improving cardiovascular risk factors, insulin sensitivity, body weight, physical fitness, lipid levels, blood pressure, and well-being, thereby reducing the risk of cardiovascular-related morbidity and mortality [40]. This study demonstrates that there is a need for intervention in the Brazilian T2DM population directed toward improving CRF and thereby preventing CVD-related morbidity and mortality.

### Strengths and Limitations 

This study explored the presence of a relationship between poor glycaemic control and inadequate levels of cardiorespiratory fitness among a Brazilian population with T2DM. Furthermore, this study found that to predict VT and VO_2_ max, variables such as HBA1c, diabetes duration, age, and BMI can be employed. However, the results should be taken with caution due to the small sample size and cross-sectional design. Moreover, there is the possibility that the presence or absence of an associated comorbid status affected the CRF measures. 

## 5. Conclusions 

The present study demonstrates the existence of poor glycaemic control and cardiorespiratory-fitness-related parameters in the Brazilian T2DM population. Moreover, this study found that poor glycaemic control may negatively impact CRF parameters. HBA1c, duration of diabetes, age, and BMI can be employed to predict VT and VO_2_ max. These results are indicative of a high risk of cardiovascular events and related complications in the T2DM population. 

## Figures and Tables

**Table 1:** Demographic details of the individuals with type 2 diabetes mellitus.

Mean (SD)	HBA1C ≤ 7	HBA1C ≥ 7.1
Age (Years)	52.5(7.3)	52.65(8.4)
Duration of diabetes (Years)	4.3(5.7)	7.9(5.2) *
Height (cm)	168.9(10.2)	167(26.3)
Weight (kg)	87.9(17.4)	83.5(19.7)
BMI	30.9(5.9)	29.4(5)
Resting PetCO_2_	37(3.3)	36.4(3.1)
VT	14.3(5.2)	13.4(3)
Peak RER	1.1(0.2)	1.0(0.1)
VO_2_ max (mL/kg/min)	22.4(5.3)	21.1(5)
Peak PetCO_2_ (mmHg)	39.8(6.4)	38.8(4.7)
Peak HR (bpm)	150.7(19.7)	151.5(17.6)
Peak SBP (mmHg)	213.9(34.1)	208.2(31.7)
Peak DBP (mmHg)	95.7(14.4)	99.8(14.9)
V_E_/VCO_2_ slope	32.2(6.7)	33.1(8)
OUES	2.14(0.6)	2.0(0.6)
Heart rate recovery in 1 min (bpm)	76.53(66.6)	73.6(60.6)
FBS (mg/dL)	123.1(31.6)	164.9(60) *
HBA1c (%)	6.15(1)	9(1.5) *

* Represents *p* value at 95% confidence interval, *p* value less than 0.05, BMI—body mass index, VT—ventilatory threshold, OUES—oxygen uptake efficiency slope, VO_2_ max—peak volume of oxygen, Peak HR—peak heart rate, Peak DBP—peak diastolic pressure, Peak RER—peak respiratory exchange rate, V_E_—minute ventilation, VCO_2_—volume of carbon dioxide output, FBS—fasting blood sugar, HBA1c (%)—glycated haemoglobin, PetCO_2_—pressure of end tidal volume of carbon dioxide (mm Hg).

**Table 2:** Pearson correlations for glycaemic parameters and cardiorespiratory fitness variables.

Variable	Pearson Correlation	*p* Value
HBA1c and duration of diabetes	0.45	<0.05
VT and FBS	0.42	0.02
V_E_/VCO_2_ and HR recovery	0.55	<0.05

HBA1c—glycated haemoglobin, VT—ventilatory threshold, FBG—fasting blood glucose, V_E_—minute ventilation, VCO_2_—volume of carbon dioxide output, HR—heart rate.

**Table 3:** Distribution of VO_2_ max according to age and gender, and risk interpretation.

Age Range (years)	VO_2_ Peak		Interpretation		Risk
	Male	Female	Male	Female	
31–40	29.5(5)	18.4(4.3)	Very poor	Very poor	High
41–50	23.3(5.7)	16.8(3.6)	Very poor	Very poor	High
51–60	23.1(4.3)	19.7(2.3)	Very poor	Poor	High
61–70	19(5)	18(0)	Very poor	Poor	High

## Data Availability

Data available on request as per the Rules and Regulation of the University.
